# Exploiting Smart Contracts for Capability-Based Access Control in the Internet of Things †

**DOI:** 10.3390/s20061793

**Published:** 2020-03-24

**Authors:** Yuta Nakamura, Yuanyu Zhang, Masahiro Sasabe, Shoji Kasahara

**Affiliations:** Graduate School of Science and Technology, Nara Institute of Science and Technology, 8916-5 Takayama-Cho, Ikoma, Nara 630-0192, Japan; nakamura.yuta.ns1@is.naist.jp (Y.N.); sasabe@is.naist.jp (M.S.); kasahara@is.naist.jp (S.K.)

**Keywords:** Ethereum Blockchain, Internet of Things, Capability-Based Access Control (CapBAC)

## Abstract

Due to the rapid penetration of the Internet of Things (IoT) into human life, illegal access to IoT resources (e.g., data and actuators) has greatly threatened our safety. Access control, which specifies who (i.e., subjects) can access what resources (i.e., objects) under what conditions, has been recognized as an effective solution to address this issue. To cope with the distributed and trust-less nature of IoT systems, we propose a decentralized and trustworthy Capability-Based Access Control (CapBAC) scheme by using the Ethereum smart contract technology. In this scheme, a smart contract is created for each object to store and manage the capability tokens (i.e., data structures recording granted access rights) assigned to the related subjects, and also to verify the ownership and validity of the tokens for access control. Different from previous schemes which manage the tokens in units of subjects, i.e., one token per subject, our scheme manages the tokens in units of access rights or actions, i.e., one token per action. Such novel management achieves more fine-grained and flexible capability delegation and also ensures the consistency between the delegation information and the information stored in the tokens. We implemented the proposed CapBAC scheme in a locally constructed Ethereum blockchain network to demonstrate its feasibility. In addition, we measured the monetary cost of our scheme in terms of gas consumption to compare our scheme with the existing Blockchain-Enabled Decentralized Capability-Based Access Control (BlendCAC) scheme proposed by other researchers. The experimental results show that the proposed scheme outperforms the BlendCAC scheme in terms of the flexibility, granularity, and consistency of capability delegation at almost the same monetary cost.

## 1. Introduction

Thanks to the maturation and commercialization of the Internet of Things (IoT), recent years have witnessed explosive growth of smart devices (e.g., appliances, wearables, and industrial equipment) connected to the Internet. It was reported that over 200 billion IoT devices will be connected to form an extremely huge IoT network by 2020 [1]. Although these devices make our lives more convenient and intelligent, they are vulnerable to illegal access by malicious users, posing significant threats to our personal and property safety [2]. For example, malicious users may know the contents of private conversations inside a home by illegally accessing some appliances [3,4,5,6]. In addition, malicious users may also be able to gain illegal access to the control unit (e.g., brake and accelerator) of a self-driving car to cause severe accidents [7]. Access control, which explicitly or implicitly specifies who (i.e., subjects) can access what resources (i.e., objects) under what conditions, has been identified as an effective solution to preventing unauthorized access [8,9]. Therefore, our research focuses on the access control issue in the IoT.

### 1.1. Access Control

#### 1.1.1. Common Models

Commonly-used access control models include Role-Based Access Control (RBAC), Attribute-Based Access Control (ABAC), and Capability-Based Access Control (CapBAC). In the RBAC model [10,11], a specific role is assigned to each subject. In addition, permissions to perform some operations on certain devices are assigned to each role. By doing this, permissions are assigned to subjects. Grouping access permissions by roles simplifies the management of access control compared to assigning access permissions to each subject individually. In the ABAC model [12,13], determining whether access is allowed or denied is based on policies, which are statements that combine the attributes of subjects, objects, actions, and dynamic context (e.g., time and location information) to achieve dynamic access control.

In the CapBAC model [14,15], each subject is associated with a capability, i.e., a token that stores the access rights of the subject. When accessing an object, each subject needs to deliver his or her token to the object owner. The owner then decides if the subject can access the object by checking the validity of the token. Two main operations in CapBAC are capability delegation and capability revocation. Delegation means that a subject delegates all or part of his/her access rights to another subject. Revocation means that a subject revokes the access rights he/she has delegated to avoid the abuse of the access rights.

#### 1.1.2. Centralized vs. Decentralized

Traditionally, centralized access control schemes, which usually rely on a central server for all the access control-related processing including access right assignment, management (e.g., update and revocation), and verification, have been the mainstream schemes in the field of access control [13,16,17]. Despite the ease of management, the server in these schemes turns out to be a single point of failure and may destroy the access control system once it suffers from man-made/natural disasters or is compromised by adversaries [18]. Besides, it is usually difficult for centralized access control schemes to cope with the large-scale and distributed nature of IoT systems [19]. Therefore, to overcome the above limitations of centralized access control schemes, research efforts have been devoted to the design of decentralized access control schemes for the IoT [20].

In decentralized access control schemes, the majority of the system nodes instead of a single server are responsible for the access control-related processing. The key to the proper operation of decentralized access control schemes is that all nodes must reach a common consensus on the data for access control such as assigned rights of subjects, access policies, and verification results. Such consensus can ensure robust and trustworthy access control and must be resistant to any tampering as well, i.e., no one can deceive others by tampering with the access control data. Recently, the emerging blockchain technology has been proved as one of the most promising mechanisms for reaching common consensuses in a distributed environment, thanks to its successful application in cryptocurrency systems, such as Bitcoin [21]. This is why there is an increasing interest in applying the blockchain technology to achieve decentralized and trustworthy access control for the IoT.

### 1.2. Blockchain and Smart Contract

**Figure 1:**
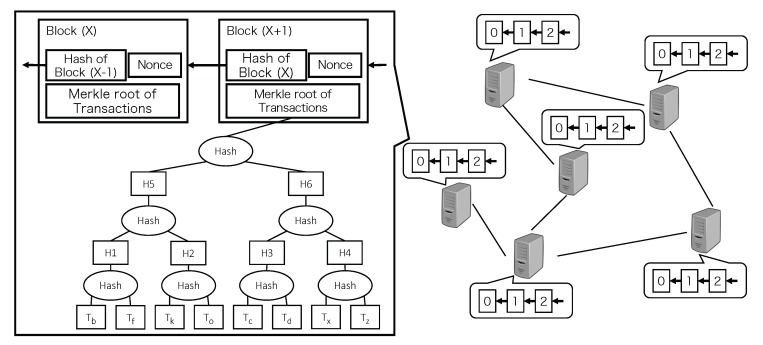
Blockchain system.

Blockchain was originally invented as a distributed and tamper-resistant ledger for cryptocurrency systems to store the financial transfer data (i.e., transactions). Figure 1 illustrates the data structure of blockchain and the blockchain system built on a Peer-to-Peer (P2P) network. The blockchain consists of a collection of blocks, and each block contains a hash of transactions (i.e., the root of Merkle tree) and the hash of its previous block. All blocks are shared by all nodes in the P2P network. The most appealing feature of the blockchain is its ability to reach consensuses on its states (e.g., transaction history and balances) among its participants by using cryptographic hash functions, even in the presence of attackers.

The common consensus is achieved based on the mining process, which is the process of generating new blocks by some special nodes called miners. In this process, each miner first collects a set of transactions from its transaction pool and generates a Merkle tree (as shown in Figure 1) using these transactions. The miner then includes the set of transactions, the Merkle tree, the hash of the previous block, and an arbitrary number called nonce into the block to generate. After including all necessary information into the block, the miner finally keeps calculating the hash of the block by varying the value of the nonce, until it finds a valid hash value that satisfies a pre-defined condition, for example, the leading *n* bits of the hash must be zeros. The miner that first finds a valid block wins in this process and this block will be broadcast to all the other nodes in the network. Each node then verifies the validity of the received block and then includes the block into its own blockchain if the block is valid. In this way, the common consensus is achieved.

**Figure 2:**
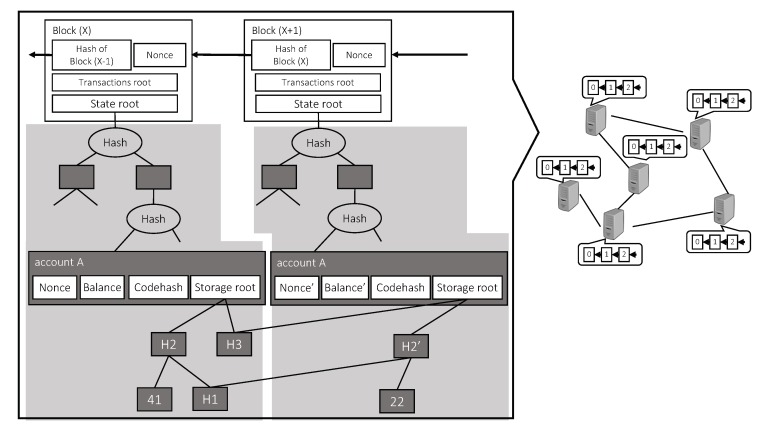
Data Structure of Ethereum Blockchain.

In addition to transactions, executable programs called smart contracts can also be stored on current blockchains such as Ethereum [22,23], transforming the blockchains from pure distributed databases to hybrid distributed storage and computing platforms. In Ethereum, smart contracts are special accounts, whose information is stored in the *State root* field of block headers. A smart contract usually consists of variables as its states and functions called Application Binary Interfaces (ABIs) to view and change the states. The ABIs are stored in the *Codehash* field (as shown in Figure 2) of the contract, which is the hash of the program code. The variables are stored by the *Storage root* field, i.e., the hash of the variables. In addition, a smart contract also contains a *Nonce* field, which records the number of smart contracts created by this contract, and a *Balance* field, which is the balance of this contract. Each ABI is usually triggered by sending a transaction to the contract to change the variables. The transaction is broadcast in the P2P network, and every node that receives the transaction will also execute the ABI to verify that the results are correct. In this way, the consensus on the states of the variables can be reached.

Thanks to its appealing features, the blockchain technology has been applied to the IoT to transform the service provision mechanism from traditional Service-oriented Architecture (SoA) to novel blockchain-based microservice architectures [24,25]. In addition, the blockchain also affects many other fields of the IoT, such as access control which is introduced in Section 2, data sharing, and business models [26].

### 1.3. Research Objective

The goal of our research is to implement distributed and trustworthy access control for the IoT using Ethereum smart contracts. In particular, we focus on the CapBAC model. Despite the drawback of low context-awareness as pointed out by the authors of [9,27], the CapBAC model can ensure the critical principle of least privilege, i.e., each subject uses the least amount of privilege (i.e., access rights) necessary to finish its job. In addition, the CapBAC model allows subjects to delegate access rights from one to another for flexible and spontaneous access control. Recently, some initial attempts have been made to implement access control using the blockchain technology [28,29,30,31,32,33,34,35,36,37,38,39,40,41,42,43,44,45]. Among these schemes, the Blockchain-Enabled Decentralized Capability-Based Access Control (BlendCAC) scheme in [28] is the one most related to our scheme. We introduce the BlendCAC scheme including its main idea and limitations as well as our contributions in Section 3. For the introduction of other schemes, please refer to the related work in Section 2.

The remainder of this paper is organized as follows. Section 4 introduces the proposed CapBAC scheme, Section 5 presents the implementation details of the proposed scheme, and Section 6 evaluates the monetary cost of the proposed scheme in terms of gas consumption and also compares the monetary cost of the proposed scheme with that of the BlendCAC scheme. Finally, we conclude this paper in Section 7.

## 2. Related Work

In [29], a Bitcoin-like blockchain was implemented to achieve access control in a smart home application based on the Access Control List (ACL) model. The authors deployed a local blockchain in each home to store the ACL that controls the access requests from inside and outside of the home. Since the blockchain is maintained only by a single miner and the critical mining process is eliminated, the access control in each home becomes centralized and untrustworthy. The authors of [30] used the Bitcoin transactions to store access policies for an existing ABAC scheme. In the ABAC model, each policy combines the attributes of subjects, objects, actions, and context to provide dynamic and fine-grained access control. When receiving an access request, the ABAC scheme retrieves the related policies from the blockchain to perform the access control. Similar to Francesco et al. [30], the Bitcoin transactions were used to store the tokens of the CapBAC model by Ouaddah et al. [31]. By sending transactions among the subjects, the capability tokens can be delegated from one subject to another. When accessing an object, the subject passes its own capability token to the object owner, who then performs the access control by checking the validity of the token. In their next study, Francesco et al. [32] considered smart contracts to enforce access control policies.

Recently, Ethereum smart contract-based access control schemes have attracted considerable attentions. In [33], an ACL-based IoT access control framework was designed using multiple smart contracts. Each contract stores an ACL and the corresponding access control ABI for one subject–object pair. The authors also provided implementations to demonstrate the feasibility of the framework. In [34], an extended version of the scheme in [33] was designed with slight modification. In [35], a smart contract was deployed to maintain the roles assigned to each user in an RBAC model, such that any service provider can verify the users’ ownership of roles when providing services. A CapBAC-like scheme was proposed in [36] to manage access control for data sharing in IoT systems, where oracles are used to connect blockchain, data hosts, and users for data accessing. Another CapBAC-like scheme was proposed in [37] for handling the access control in information-centric networks. An ABAC scheme was proposed in [38], which stores the URL links of policies on the blockchain and also deploys a smart contract for access control. When accessing an object, a subject sends the link of the related policy to the smart contract, which then retrieves the policy from external databases to achieve the access control. However, adversaries may be able to tamper with the polices without changing the URL links, resulting in untrustworthy access control. To address this issue, a novel ABAC framework was proposed in [39], which directly stores the policies as well as the attributes of subjects and objects on the blockchain. A similar idea was adopted in [40,41] but with different realizations.

Access control based on other blockchain realizations has also been investigated. For example, in [42], the authors proposed an ABAC framework based on the permissioned Hyperledger Fabric blockchain, while, different from Yutaka et al. [39], only the attributes are stored on the blockchain and no smart contracts are used for processing access requests. By integrating attribute-based encryption with blockchain, the authors of [43] proposed another ABAC-like scheme based on a multi-layer blockchain architecture. A conceptual design of blockchain-based ABAC scheme was provided in [44], while the authors presented no implementations.

In addition to access control, there also exist some other alternative methods that can prevent unauthorized access to some extent, such as authentication [46], which deals with authenticating the identity of resource users, and intrusion detection [47], which prevents unauthorized users from entering IoT systems. These methods are usually combined with access control to provide full protection to IoT resources.

## 3. BlendCAC Scheme

To manage the authorized actions (i.e., access rights) of the subjects for each object, the BlendCAC scheme defines two types of tokens: Identity-based Capability (ICap) and Identity-based Delegation Certificate (IDC). An ICap token records the authorized actions (e.g., read, write, and execute) of a subject and an IDC token records the delegation relationships of the authorized actions among the subjects. The following expressions illustrate the data structures of an ICap token and an IDC token of a certain subject *S*, respectively.

(1) $$\begin{array}{c} ICap_{O}\left[VID_{S}\right]=\left\{OP\right\}, \end{array}$$

(2) $$\begin{array}{c} IDC_{O}\left[VID_{S}\right]=\left\{VID_{P},\left\{VID_{Ch}\right\},Dep\right\}, \end{array}$$

where the meanings of the symbols are described in Table 1.

**Figure 3:**
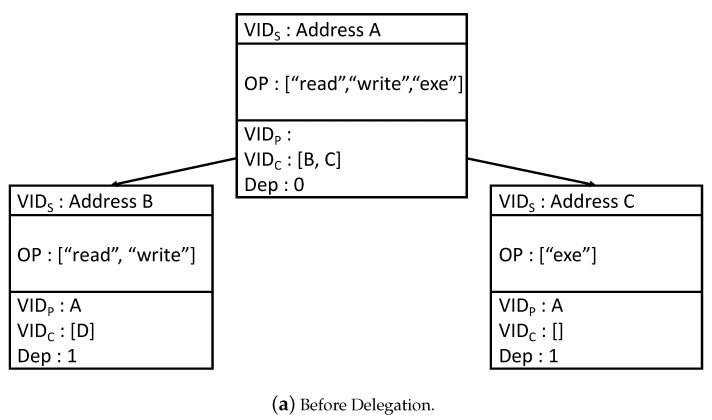

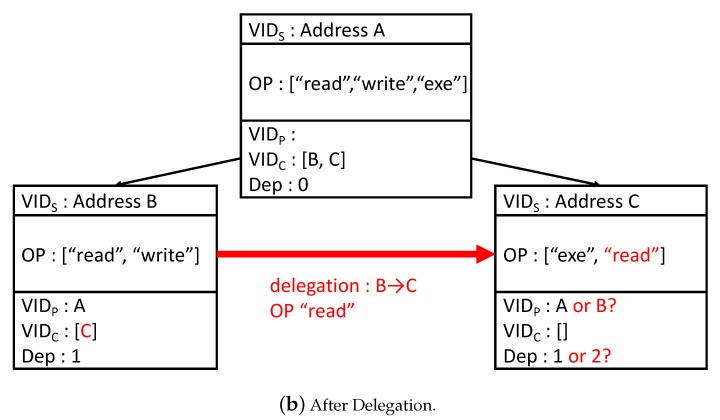
Delegation Tree in the BlendCAC Scheme.

Using these tokens, the BlendCAC scheme manages the capabilities of subjects and their delegation relationships for each object by a delegation tree. Figure 3 shows an example of the delegation tree with three subjects *A*, *B*, and *C*. This tree shows that subject *A*, the owner of the object, delegates its *read* and *write* rights to subject *B* and *exe* (i.e., execute) right to subject *C*. The parent subjects of *B* and *C* are set as *A* due to the delegation. Consider now the case where *B* needs to delegate its *read* right to *C*. In this case, should *A* or *B* be the parent subject of *C*? We can see that neither *A* nor *B* as the parent subject can record all the delegation information completely. A similar problem arises to the *Dep* information. As a result, a subject cannot obtain rights from more than one subject due to the contradiction/ambiguity about the delegation information. In addition, to complete a delegation, the related ICap and IDC tokens must be updated synchronously. However, this requirement cannot always been satisfied in the blockchain system, due to the difference between the time instants when the two transactions for updating the tokens are included into the blockchain.

To address the above limitations of the BlendCAC scheme, we propose a novel smart contract-based CapBAC scheme with more fine-grained capability management and more flexible capability delegation. More specifically, we first define the capability tokens *in units of authorized actions*, i.e., in the manner of one token per action instead of one token per subject as in the BlendCAC scheme. Second, we use *one type of token* to summarize the information of capabilities and delegation relationship so as to update these information simultaneously. Finally, we manage the delegation relationship of the subjects by a *delegation graph* instead of the delegation tree in the BlendCAC scheme to enable more flexible capability delegation. Compared with the BlendCAC scheme, the proposed scheme also provides the functionality of adding new authorized actions. A conference version of this paper can be found in [48].

## 4. Proposed CapBAC Scheme

This section introduces the proposed CapBAC scheme including the structure of the capability token, the delegation graph, and the main functions.

### 4.1. Capability Token Structure and Delegation Graph

We first revise the capability token structure by splitting the capability tokens of the BlendCAC scheme into multiple ones based on the authorized actions with each being associated with an action. Thus, each token can uniquely be identified by the ID of the subject $VID_{S}$ and an action OP, as shown in the following expression.

(3) $$\begin{array}{c} CAPS_{O}\left[VID_{S}\right]\left[OP\right]\;\;=\;\;\left\{VID_{P},\left\{VID_{Ch}\right\},Dep,DR,RR\right\}, \end{array}$$

where the meanings of *O*, *C*, $VID_{P}$, $\left\{VID_{Ch}\right\}$, and Dep are described in Table 1.

Note that our scheme uses the Ethereum account addresses as the ID information of both subjects and objects. The field DR indicates whether the owner of the token (i.e., $VID_{S}$) can further delegate it to other subjects. Similarly, the field RR indicates whether the subject $VID_{S}$ can revoke the delegated tokens from the descendant subjects in $\left\{VID_{Ch}\right\}$. This structure allows each subject to own multiple tokens and to flexibly delegate authorized actions to and from multiple subjects. In addition, we can use only one type of tokens for each object to manage the capabilities of the subjects and construct a delegation graph for managing the delegation relationships.

**Figure 4:**
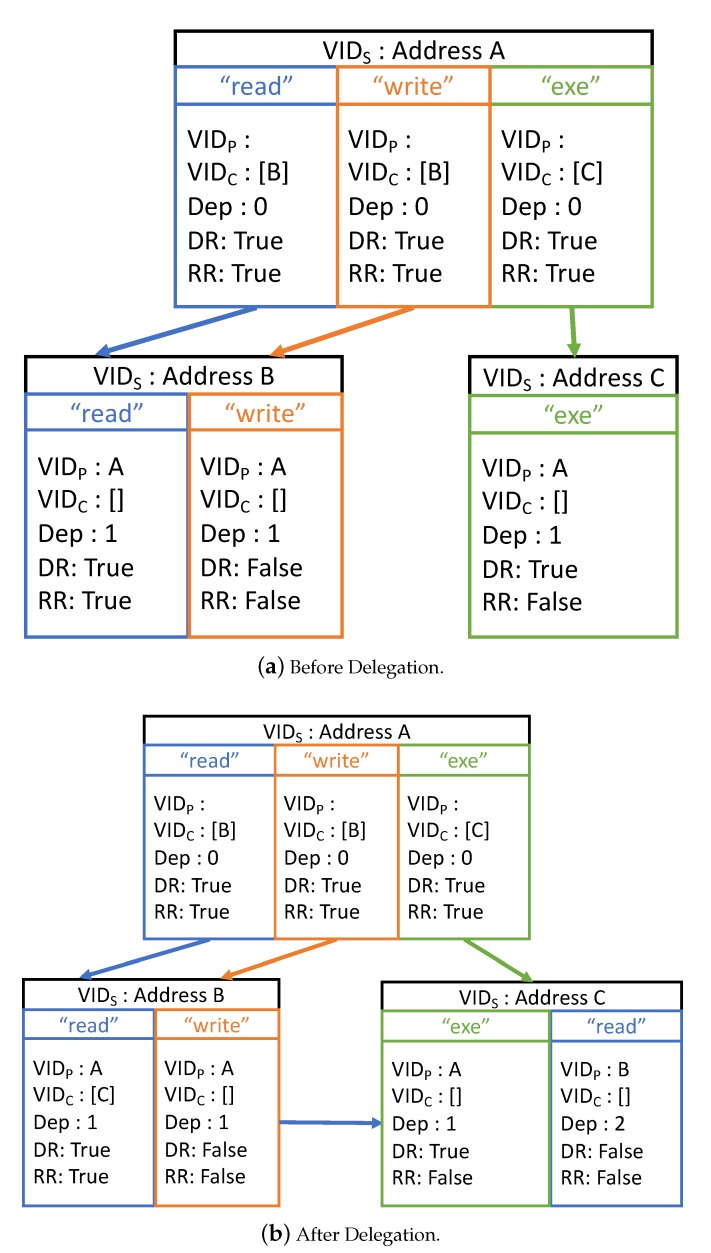
Delegation Graph of the Proposed CapBAC Scheme.

Figure 4 illustrates a simple example of the delegation graph for an object *O* with three subjects *A*, *B*, and *C*. Subject *A*, the owner of the object, has three tokens with authorized actions *read*, *write*, and *exe*, and delegates the *read* and *write* tokens (respectively, *exe* token) to subject *B* (respectively, *C*). Again, we consider the case where *B* needs to delegate its *read* token to *C*. Because each token is independent of the others, the delegation causes no contradiction or ambiguity about the delegation information. After the delegation, *C* is appended to the set of descendant subjects (i.e., $\left\{VID_{Ch}\right\}$) of the *read* action of *B*, and *B* becomes the parent subject of *C* in terms of the *read* action. Accordingly, the depth of the delegated *read* token of *C* is increased by 1 compared with that of *B*. To manage the tokens and delegation graph, we deploy a smart contract on the Ethereum blockchain, the main functions of which are described in the following section.

### 4.2. Main Functions

The proposed CapBAC scheme provides the following main functions including token creation, token delegation, token revocation, and token verification, which are introduced as follows.

#### 4.2.1. Token Creation

Different objects require different sets of authorized actions. When the current set of actions is not enough to support new applications, some new actions may be needed. In this case, the function of token creation can be used. The smart contract provides a *createAction()* ABI for this function. Only the owner of the objects has permissions to execute this ABI. When executing this ABI, the owner needs to send a transaction containing the information defined in (3).

#### 4.2.2. Token Delegation

Token delegation is a fundamental and critical function of CapBAC schemes to support flexible and spontaneous access. Subjects can gain access rights through the tokens delegated by other subjects without the intervention of the owner, improving the scalability of the access control scheme. The smart contract provides a *delegation()* ABI to enable the token delegation. Only the owner of the token can execute this ABI by sending a transaction with the required information.

#### 4.2.3. Token Revocation

When the delegator (i.e., the subject that delegates the token) of a token decides that the current owner no longer has access permissions, it can revoke the token to avoid token abuse. The smart contract provides two ABIs, i.e., *singleRevocation()* and *allChildrenRevocation()*, to support the token revocation. The *singleRevocation()* ABI revokes the tokens from the children of the delegator, while the *allChildrenRevocation()* ABI revokes the tokens from all the descendants.

#### 4.2.4. Token Verification

When accessing an object, a subject needs to hand the related token to the object’s owner, which then performs the token verification to confirm that the subject has the required access rights. The smart contract provides an *accessRequest()* ABI for the token verification. Any subject can execute this ABI by offering the required information such as the subject’s ID and the action to perform via a transaction. The transaction will be mined, included into a block and broadcast to most of the nodes in the system. During this process, each node that receives this transaction will execute the ABI to confirm whether the subject has the required access rights. This ensures that no nodes can deceive others with wrong processing results, achieving robust and trustworthy access control. After the verification of the token, the results will be returned to both the subject and the object.

## 5. Implementation

In this section, we implement the capability, delegation graph, and functions introduced in Section 4 to demonstrate the feasibility of the proposed CapBAC scheme [49].

### 5.1. Ethereum Private Network

**Figure 5:**
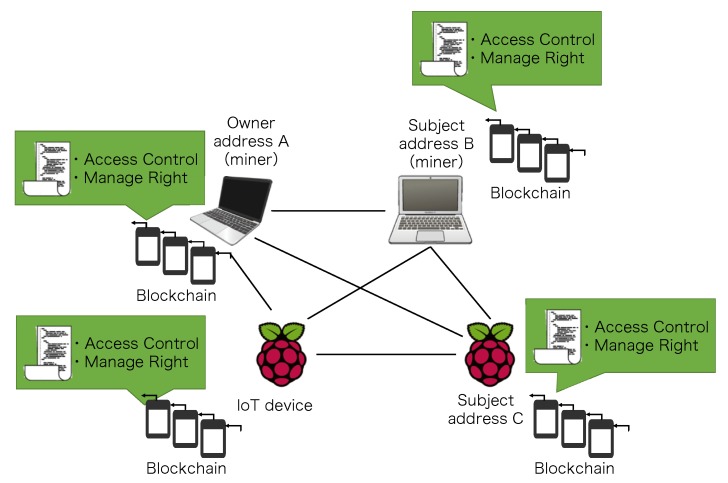
Experiment Environment.

As shown in Figure 5, we built a private Ethereum blockchain network using one MacBook Pro (CPU: 3.1 GHz Intel Core i5, Memory: 8 GB), one MacBook Air (CPU: 1.8 GHz Intel Core i5, Memory: 8 GB) and two Raspberry Pis (CPU: 1.4 GHz ARM Cortex-A, Memory: 1 GB). One Pi works as the object and the other works as the subject. The MacBook Pro plays the role of the owner entity of the object. To form a private Ethereum blockchain network, each device maintains a local copy of the blockchain and interacts with the blockchain (e.g., send transaction and obtain access result) through a JavaScript program based on the web3.js package [50]. The MacBook Pro and MacBook Air serve as miners in this private Ethereum blockchain network. We created Ethereum accounts addressA, addressB, and addressC for the MacBook Pro, MacBook Air, and the subject Raspberry Pi, respectively, the information of which is summarized in Table 2.

### 5.2. Token Creation

**Figure 6:**
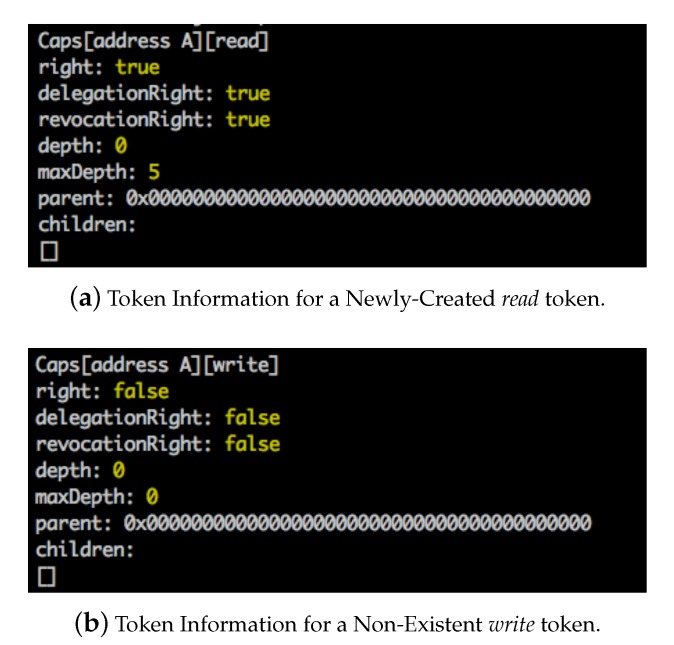
Token Creation by the Owner Entity addressA.

At the beginning of the experiment, the owner entity with address addressA registered a smart contract to store and manage the capability tokens and the delegation graph. The owner entity then created new tokens by executing the *createAction()* ABI. Figure 6a shows the information returned by calling the *getCap()* ABI via the javaScript program after the owner entity created a new *read* token for the subject addressA (i.e., the owner entity). The token states that the subject addressA has *read* right to the object. It can also delegate this token to other objects and revoke the token from its descendants. At this point, the token has depth (depth=0) and maximum depth (maxDepth=5), which means that the token can be further delegated to at most five generations. In addition, we can see that the token has no parents and no children. Figure 6b shows the result of calling the *getCap()* ABI to query a token (i.e., the *write* token) that does not exist or has not been created on the blockchain. We can see that all fields are set as “empty,” since the javaScript program cannot fetch any information from the blockchain.

### 5.3. Token Delegation

**Figure 7:**
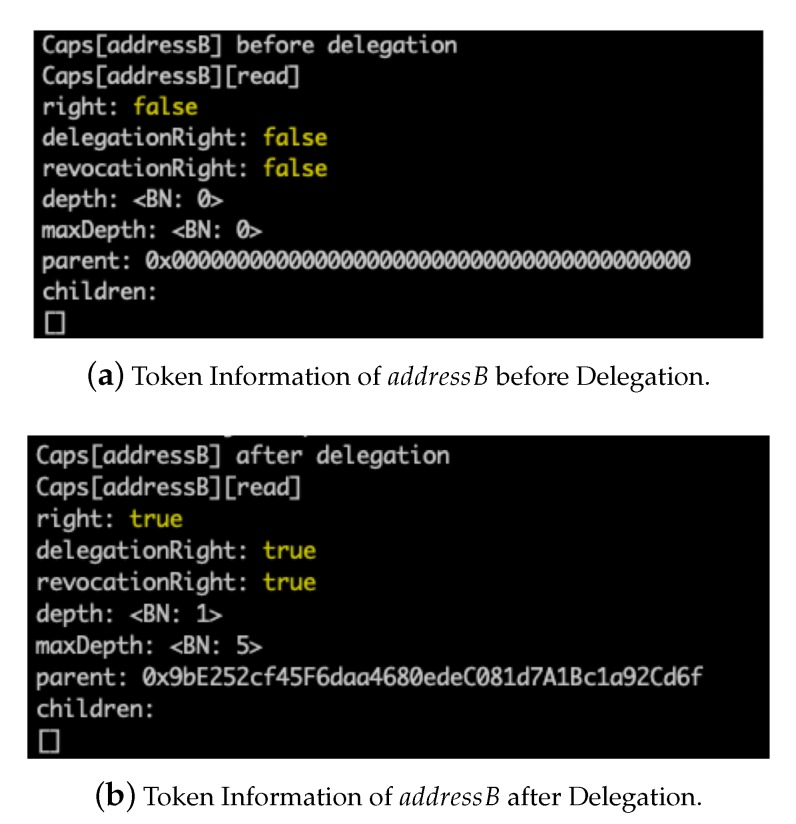
Delegation of *read* Token from Subjects addressA to addressB.

After creating new tokens, the owner entity addressA then delegated the tokens to other subjects. Figure 7a,b show the token information of the subject subjectB before and after the owner entity delegates the *read* token to it, respectively. We can see that the *right* field of the *read* token changes from *false* to *true* after the delegation, which means that the delegation successfully delivers the *read* right to the subject addressB. In addition, the *depth*, *maxDepth*, and *parent* fields are changed accordingly. Note that the fields of “*delegationRight: true*” and “*revocationRight: true*” indicate that the subject addressB can delegate the token to other subjects and revoke the token when necessary.

### 5.4. Token Revocation

**Figure 8:**
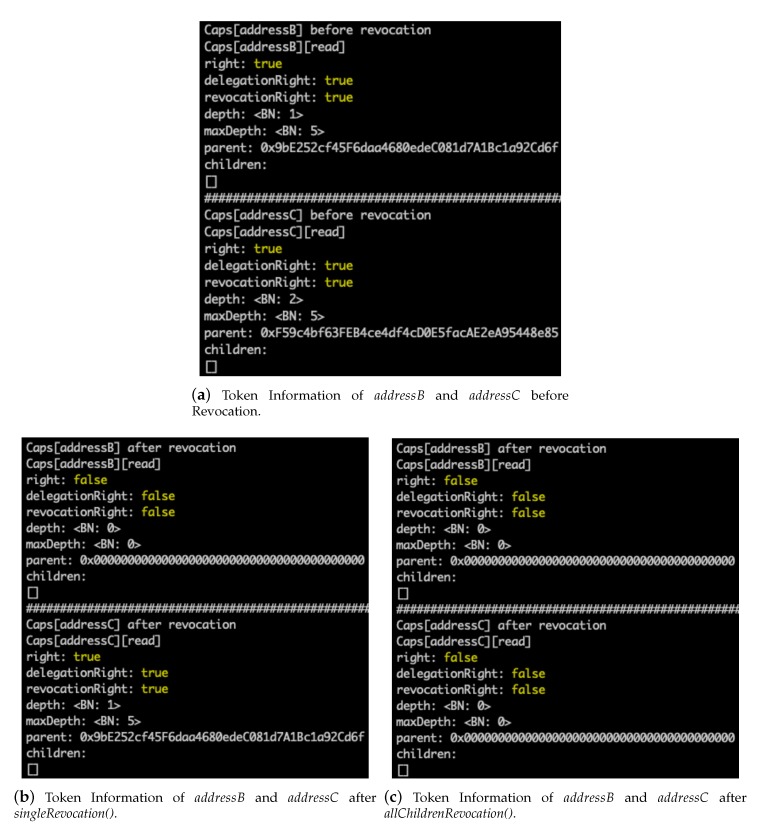
Revocation of *read* Token.

Suppose that the subject addressB further delegated the *read* token to another subject addressC. At this point, the token information of the subjects addressB and addressC is shown in Figure 8a. When the token delegator (i.e., subject addressA) decides to revoke the token from the subject addressB but allow the subject addressC to keep the token, the delegator executes the *singleRevocation()* ABI. Figure 8b shows the token information after the execution of the ABI, where only the token information of subject addressB is deleted. Note that the parent of subject addressC is changed from addressB to addressA, and thus the depth is also decreased by one. On the other hand, if the delegator wants to revoke the tokens from both subjects addressB and addressC, the delegator executes the *allChildrenRevocation()* ABI. Figure 8c shows the token information after the execution of the ABI, where the token information of both subjects is deleted.

### 5.5. Token Verification

**Figure 9:**
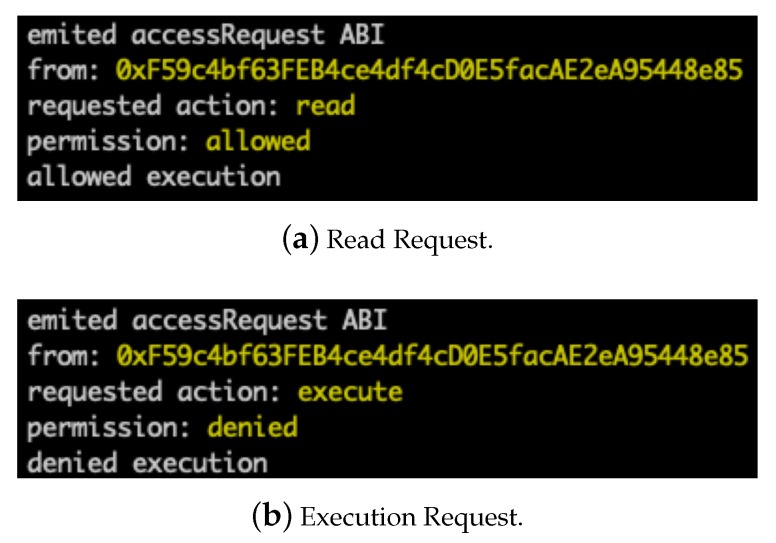
Access Result When Subject addressB Sends Requests.

After receiving the *read* token, the subject addressB can pass the token to the smart contract to verify that it has the *read* right when it wants to read the object. Figure 9a depicts the result when the subject addressB sent the read request. The result shows that the subject addressB is allowed to read the object. For comparison, Figure 9b depicts a rejected execution request when the subject addressB does not own the corresponding token.

## 6. Monetary Cost Evaluation

The Ethereum blockchain system requires users to pay some fee for issuing a transaction. This fee will be paid to the miner who includes the transaction into the winning block, i.e., the block created by the winner of the mining competition. Note that users usually need to issue a transaction to execute an ABI of a smart contract. This means that users have to pay some transaction fee for using the functions provided by our access control system. Ethereum uses *Ether* as the unit for measuring the transaction fee, which is about 160 US dollars as of 10:00 JST, January 16, 2020.

In general, the transaction fee TxFee of calling an ABI can be expressed in the following equation [23].

(4) TxFee=gas×gasPrice,

where gas is Ethereum’s unit for measuring the computing and storage resources required to perform the actions of ABIs, and gasPrice, measured in *Ether/gas*, is the price for each unit of gas.

When issuing a transaction, the sender needs to specify the gas price he/she wants to pay for the transaction. Because the total gas amount of transactions included in a block cannot be larger than 8 million (called gas limit), miners prefer to including transactions with higher gas price into their blocks to gain more reward. Consequently, transactions with higher gas price are faster to appear in the blockchain in general.

Because the gas price varies with senders, this study measured the amount of gas instead of Ether used by each ABI and used this amount as the metric for evaluating the proposed access control scheme. In addition, to compare our scheme with the BlendCAC scheme in [28] in terms of the gas cost, we also measured the amount of gas consumed by the ABIs of the latter, including *createAction()*, *delegation()*, and *allChildrenRevocation()*. Table 3 shows the variables used in the measurement of the gas cost.

### 6.1. Token Creation

When the owners of an object create a token, they need to send a transaction specifying the new token’s action name. The length of the action name affects the storage resources and gas used by the *createAction()* ABI. Thus, we consider three patterns of the input action name, namely *pattern1*, *pattern2*, and *pattern3*, with lengths of 3, 4, and 5, respectively. Table 4 shows some examples of the three patterns as well as the amount of gas used by each example.

We can see that the first token creation of each pattern uses about 15,000 more gas than the subsequent ones. This is because the *createAction()* ABI needs to initially allocate some memory for tokens and the subject address $VID_{S}$, which consumes some extra gas. By comparing the three patterns, we can observe that the amount of used gas increases by 64 as the length of the action name increases by one character. The relationship between the amount of used gas and the length of input action names can be expressed by

(5) $$\begin{array}{cc} gas & =108710+64\times len\left(Act\right)+15000\times 1_{first\;time}, \end{array}$$

where $1_{first\;time}$ is an indicator function, which equals 1 for the first time of token creation. We can also observe that the gas consumption of the token creation is independent of the number of tokens created since the second creation, i.e., remains constant as the latter increases. In the following measurements, we set the length of the action name to 4.

In the BlendCAC scheme, the OP’s (i.e., actions) are managed by an array. Thus, as the number of OP’s increases, a larger array is needed and more computing resource will be used as well. Table 5 shows the relationship between the amount of gas and the length of the OP array (i.e., len(Op[])), which can be expressed by

(6) $$\begin{array}{cc} gas & =49438+len\left(Op\left[\right]\right)\times 2336+14997\times 1_{first\;time}, \end{array}$$

Comparing the results in Table 5 and the *pattern2* column of Table 4, we can observe that when the number of actions is small, the creation of one token by the proposed scheme usually consumes more gas than that of the BlendCAC scheme (see the token "read" in both tables for an example). This is because the tokens in the proposed scheme contain more information than those in the BlendCAC scheme (i.e., contain not only the information for access control but also the relationship of delegation), thus requiring more gas. However, as the number of actions increases above a threshold, the creation of one token by the proposed scheme consumes less gas than that of the BlendCAC scheme. This is because the gas cost of token creation of the BlendCAC scheme increases almost linearly as the number of actions (i.e., the length of the OP array len(Op[])) increases, while that of the proposed scheme remains constant.

### 6.2. Token Delegation

When the *delegation()* ABI is called by the delegator to delegate a token to a delegatee, the ABI creates a token associated with the delegatee’s address and then stores the delegatee’s address in the $VID_{Ch}$ filed of the delegator’s token. Because the storage of a token is fixed in size and so is the address, it can be predicted that the *delegation()* ABI consumes a constant amount of gas. We measured the amount of gas used by the *delegation()* ABI and summarize the results in Table 6. In this measurement, a token is delegated from addressA to addressB, then from addressB to addressC, and finally from addressC to addressD. In addition, the token of addressC is further delegated to addressE and addressF, respectively. The amount of gas used for each delegation in the first case is 162,386, and that in the second case is 147,386. The difference of 15,000 gas is due to the initial cost for allocating storage for the $VID_{Ch}$ field of the token of addressC. That is, when addressC delegates its token to addressD, 15,000 gas is required to create the $VID_{Ch}$ field, while when addressC delegates its token to addressE and addressF afterwards, the $VID_{Ch}$ field already exists and thus no gas is required. The amount of gas consumed by the *delegation()* ABI can be expressed by

(7) $$gas=147386+15000\times 1_{first\;delegation},$$

where $1_{first\;delegation}$ is the indicator function, which equals 1 if the delegator delegates its tokens for the first time. Equation (7) indicates that the token delegation consumes a constant amount of gas.

Table 7 shows the gas consumption of the same delegations using the ABI of the BlendCAC scheme.

The amount of gas consumed by the delegation of the BlendCAC scheme can be expressed by the following equation:

(8) $$\begin{array}{c} gas=156509+15000\times 1_{first\;delegation}, \end{array}$$

which shows similar gas consumption to the token delegation of the proposed scheme. However, by comparing Equations (7) and (8), we can see that the token delegation of the BlendCAC scheme consumes more gas than that of the proposed scheme. This is because, when delegating a token, the former needs to modify the states of two tokens, i.e., the capability token (ICap) and the delegation certificate token (IDC).

### 6.3. Token Revocation

When the *singleRevocation()* ABI is called by the delegator to revoke a token specified by the input address, it performs three tasks: (1) rewriting the $VID_{Ch}$ filed of the revoked token’s parent token and also the $VID_{P}$ field of the revoked token’s children token; (2) deleting the revoked token from the storage; and (3) decrementing the dep field of each descendant token of the revoked token by one. Tasks (1) and (2) are expected to consume a constant amount of gas because of the fixed storage size of addresses and the token, while the amount of gas used by Task (3) is expected to be dependent on the number of the descendant tokens. We evaluated the gas consumption of the *singleRevocation()* ABI when the number of the descendant tokens is 0, 1, 2, 3, and 4, respectively. Table 8 shows the results, from which we can find the following relationship between the amount of gas used by the *singleRevocation()* ABI and the number of the descendant tokens Des:

(9) $$\begin{array}{c} gas=\left\{\begin{array}{cc} 40,329, & Des=0,\\ 60,540+(4,119\times Des), & otherwise. \end{array}\right. \end{array}$$

The above expression shows that the amount of gas used by the *singleRevocation()* ABI increases almost linearly as the number of the descendant tokens increases.

When the *allChildrenRevocation()* ABI is called to revoke all the descendant tokens, two tasks will be performed: (1) deleting a specified address from the $VID_{Ch}$ field of the parent token; and (2) deleting the token at the specified address and all the descendant tokens. Similar to the *singleRevocation()* ABI, Task (1) is expected to consume a constant amount of gas, while the amount of gas used by Task (2) depends on the number of descendant tokens. We measured the gas consumption of the *allChildrenRevocation()* ABI for the cases with 0, 1, 2, 3, and 4 descendant tokens, respectively.

Table 9 summarizes the results, from which we deduce the following linear relationship between the amount of gas used by the *allChildrenRevocation()* ABI and the number of descendant tokens Des:

(10) gas=37021+(22106×Des).

Note that only the *allChildrenRevocation()* ABI is provided by the BlendCAC scheme, thus we compare the gas consumption of the token delegation of both schemes based on this ABI. Table 10 shows the gas consumption by the *allChildrenRevocation()* ABI of the BlendCAC scheme.

Similarly, we have the following relationship between the gas consumption and number of descendant tokens Des:

(11) gas=19147×Des+32009.

The comparison between Table 9 and Table 10 shows that our scheme consumes more gas than the BlendCAC scheme in terms of the revocation of all the descendant tokens. This is because the amount of data to be deleted has increased by managing the capability tokens in units of actions.

Table 11 compares the asymptotic gas consumption of the functions provided by the proposed scheme and the BlendCAC scheme, when the number of descendant tokens Des becomes extremely large, e.g., when they are applied in a large-scale IoT system. The results show that the proposed scheme consumes less gas than the BlendCAC scheme in terms of the token creation, and has the same order of magnitude of gas consumption in terms of token delegation and token revocation from all descendants. It should be noted that the BlendCAC scheme can achieve only simple delegation but our scheme can also provide flexible delegation in the same ABI. In addition, the proposed scheme provides a new function, i.e., single token revocation.

## 7. Conclusions

In this paper, we focus on the critical access control issue for IoT resources. In particular, we aim to solve the problems of delegation ambiguity and inconsistency existing in a blockchain-based IoT access control scheme called BlendCAC proposed by other researchers, to provide more fine-grained and flexible access control. This paper proposes a CapBAC scheme by using Ethereum smart contracts to store and manage the capability tokens. Compared with the existing BlendCAC scheme, this scheme achieves more fine-grained access control and more flexible token management by defining capability tokens in units of actions and using a delegation graph to store the token delegation relationship among the subjects. Experiments based on a local Ethereum blockchain were conducted and the results demonstrate the feasibility of the scheme. We also conducted experiments to evaluate the monetary cost of the proposed scheme in terms of gas consumption. The experimental results show that the proposed scheme consumes no more gas than the BlendCAC scheme. However, low privacy of access rights is the main drawback of the proposed scheme, since the tokens are stored in the blockchain without being encrypted. Thus, one of our future works is to address this issue. Another limitation of this paper is that we fail to implement the proposed scheme in real-world IoT systems and prove that it can fulfill the security goals. We will consider these issues in our future work.

## Figures and Tables

**Table 1:** Symbols in ICap and IDC Tokens.

Variable	Meaning
*O*	The associated object.
$VID_{S}$	Identifier (ID) of the subject *S*.
OP	A set of authorized actions (e.g., read, write, and execute).
$VID_{P}$	ID of the parent subject that delegated the authorized actions to $VID_{s}$.
$\left\{VID_{Ch}\right\}$	ID of descendant subjects to which the $VID_{s}$ delegates part or all of the authorized actions.
Dep	Depth of the IDC in the delegation tree.

**Table 2:** Ethereum Addresses of the Subjects.

Variable	Address
addressA	0x9bE252cf45F6daa4680edeC081d7A1Bc1a92Cd6f
addressB	0xF59c4bf63FEB4ce4df4cD0E5facAE2eA95448e85
addressC	0x28bBa96539A24a98b3e0e3d00F4C02e201c3b080

**Table 3:** Variables in Measurement of Gas.

Variable	Meaning
len()	The number of characters or the array element.
Act	Specified action name by transaction sender.
Op[]	The array of actions used in BlendCAC.
Des	The number of the descendants of the specified address.

**Table 4:** Gas Used in *createAction()* ABI.

*Pattern1*		*Pattern2*		*Pattern3*	
*Act*	*Gas*	*Act*	*Gas*	*Act*	*Gas*
“exe”	123,902	“read”	123,966	“write”	124,030
“end”	108,902	“Edit”	108,966	“start”	109,030
“GET”	108,902	“POST”	108,966	“read2”	109,030

**Table 5:** Gas Used in BlendCAC’s *createAction()* ABI.

len(Op[])	*Act*	*Gas*
0	“read”	64,435
1	“edit”	51,774
2	“POST”	54,110
3	“exe2”	56,446

**Table 6:** Gas Used in *delegation()* ABI.

Delegator	Delegatee	Gas
addressA	addressB	162,386
addressB	addressC	162,386
addressC	addressD	162,386
addressC	addressE	147,386
addressC	addressF	147,386

**Table 7:** Gas Used in BlendCAC’s *delegation()* ABI.

Delegator	Delegatee	Gas
addressA	addressB	171,509
addressB	addressC	171,509
addressC	addressD	171,509
addressC	addressE	156,509
addressC	addressF	156,509

**Table 8:** Gas Used in *singleRevocation()* ABI.

Des	Gas
0	40,329
1	64,659
2	68,778
3	72,897
4	77,016

**Table 9:** Gas Used in *allChildrenRevocation()* ABI.

Des	Gas
0	37,021
1	59,127
2	81,233
3	103,339
4	125,445

**Table 10:** Gas Used in BlendCAC’s *allChildrenRevocation()* ABI.

Des	Gas
0	32,009
1	51,156
2	70,303
3	89,450
4	108,597

**Table 11:** The gas used in ABIs.

Functions	Our Scheme	BlendCAC
Token creation	O(1)	O(len(Op[]))
Simple delegation	O(1)	O(1)
Flexible delegation		-
All children revocation	O(Des)	O(Des)
Single revocation	O(Des)	-
